# Identification of Anthrax as the Cause of a Cluster of Unexplained Deaths, Uganda, 2023: The Role of Metagenomic Next-Generation Sequencing and Postmortem Specimens

**DOI:** 10.4269/ajtmh.24-0489

**Published:** 2025-01-07

**Authors:** Nicholas Bbosa, Deogratius Ssemwanga, Sonja L. Weiss, Sam Kalungi, Anatoli Mawanda, Richard Ssentudde, Emmanuel Ssekyeru, Alfred Ssekagiri, Ronald Kiiza, Cleophous Rwankindo, Joshua Buule, Hamidah Suubi Namagembe, Stella Nabirye, Justine Priscilla Nassolo, Robert Downing, Julius Lutwama, Tom Lutalo, Henry Kyobe Bosa, Michael G. Berg, Mary A. Rodgers, Francisco Averhoff, Gavin A. Cloherty, Pontiano Kaleebu

**Affiliations:** ^1^ MRC/UVRI & LSHTM Uganda Research Unit, Entebbe, Uganda;; ^2^ Uganda Virus Research Institute, Entebbe, Uganda;; ^3^ Abbott Pandemic Defense Coalition (APDC), Abbott Park, Illinois;; ^4^ Abbott Diagnostics, Abbott Park, Illinois;; ^5^ Pathology Department, Mulago National Referral Hospital, Kampala, Uganda;; ^6^ Ministry of Health, Kampala, Uganda;; ^7^ Kalisizo General Hospital, Kalisizo, Uganda;; ^8^ Uganda Peoples Defence Forces, Kampala, Uganda;; ^9^ Makerere University Lung Institute, Kampala, Uganda

## Abstract

Between April and November 2023, 27 unexplained human deaths that presented with swelling of the arms, skin sores with black centers, difficulty in breathing, obstructed swallowing, headaches, and other body aches were reported in Kyotera District, Uganda by the Public Health Emergency Operations Center. Subsequently, the death of cattle on farms and the consumption of carcass meat by some residents were also reported. Field response teams collected clinical/epidemiological data and autopsy samples to determine the cause of deaths. Metagenomic next-generation sequencing (mNGS) and target enrichment sequencing conducted on postmortem samples confirmed *Bacillus anthracis*, the etiological agent of anthrax disease, as the cause of the deaths. Applying mNGS to autopsy specimens is useful as a retrospective tool for identifying high-consequence pathogens during suspected outbreaks of unknown etiology.

## INTRODUCTION

Anthrax is a potentially fatal zoonotic disease caused by the bacterium *Bacillus anthracis*.^1^ Herbivores are commonly affected, and humans are incidental hosts who are often infected by exposure to contaminated soil (the spores can be stable in the environment for decades) or by direct animal contact.^2^ Anthrax presents in three forms after exposure to *B. anthracis* endospores: through abrasions in the skin, by inhalation, or by ingestion.^3^ Cutaneous anthrax is considered an occupational disease of farmers, and once the spores enter abrasions, a pruritic papule resembling an insect bite appears within 2–7 days.^4^ Gastrointestinal anthrax disease can develop if spores are ingested, mimicking gastroenteritis in the early stages and necrotizing enteritis in more severe cases.^4^ Inhalation anthrax is caused by spore inhalation, leading to acute respiratory failure and acute respiratory distress syndrome.^3^ Anthrax has the potential to be used as a bioterrorism weapon^2^^,^^5^ and was classified as a Category A bioterrorism pathogen by CDC.^6^ It can affect large populations (for instance, during the accidental anthrax release in Sverdlovsk, Russia^7^) or can be targeted deliberately, such as the terrorist attack in 2001 that resulted in 22 cases and five deaths when *B. anthracis* spores were intentionally distributed through the U.S. postal system.^5^

**Figure 1.**
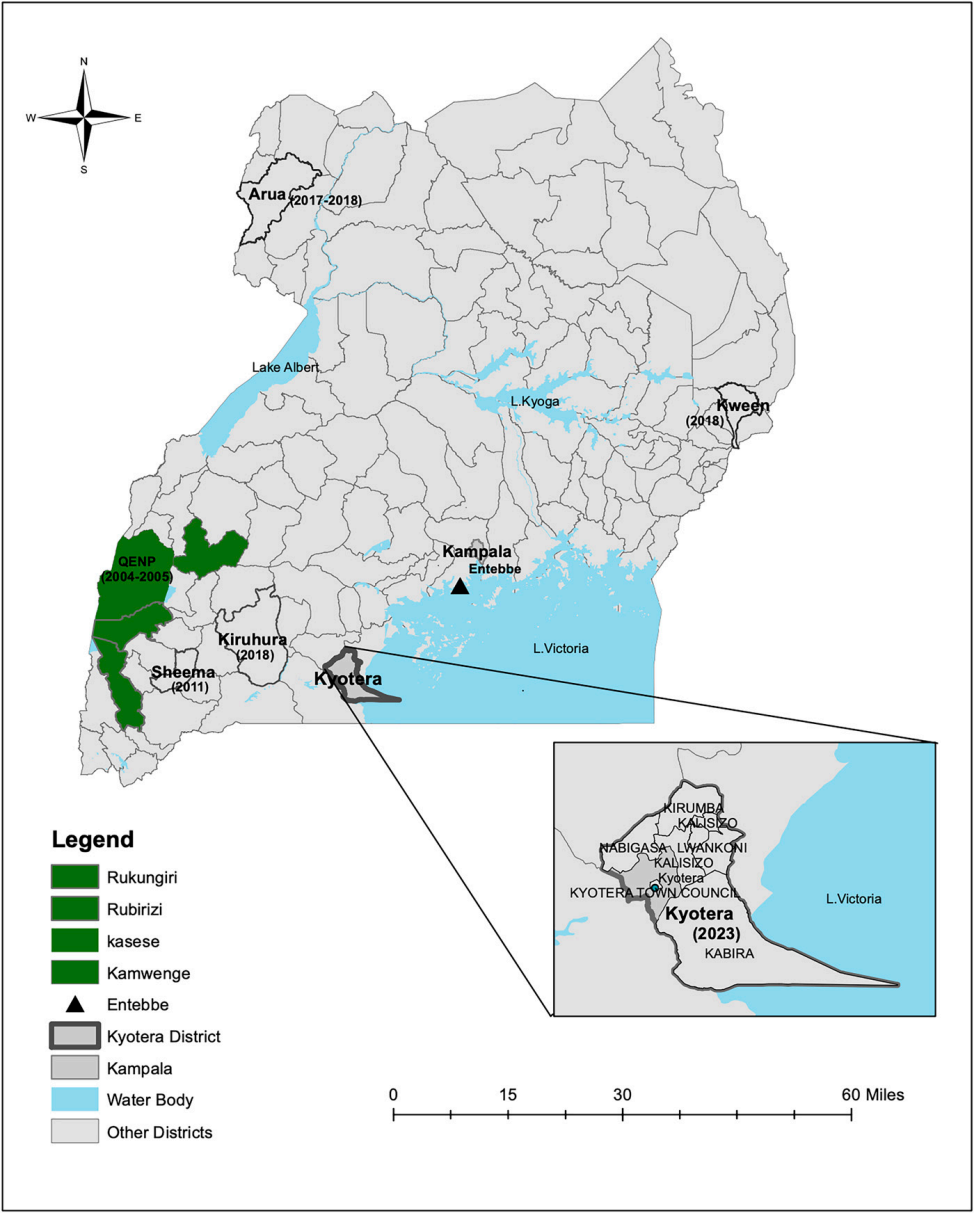
Map showing Kyotera District, where anthrax was recently confirmed by metagenomic next-generation sequencing on postmortem samples in 2023. Other districts where anthrax outbreaks have previously been reported over the years are also highlighted. These include Arua, Kween, Kiruhura, Sheema, and that districts that cover Queen Elizabeth National Park (QENP) (Kasese, Kamwenge, Rubirizi, and Rukungiri).

Globally, it is estimated that 1.1 billion livestock and 1.83 billion people live in regions where they are at risk of acquiring anthrax.^8^ In Uganda, anthrax outbreaks occur episodically^9^ and have been reported in different parts of the country (Figure 1). Between 2004 and 2005, an anthrax outbreak occurred in the Queen Elizabeth National Park (QENP) that killed 499 animals; this was followed by another outbreak in 2011 in Sheema District (western Uganda) more than 50 km from QENP.^9^ Between 2015 and 2018, other outbreaks occurred in Arua District (northern Uganda) that resulted in human cases and deaths.^10^^–^^12^ In 2018, more outbreaks were reported in Kween (eastern Uganda)^13^ and Kiruhura District (western Uganda).^14^ In these outbreaks, anthrax disease was identified either by rapid diagnostic tests or gram/M’Fadyean staining ^10^ or by polymerase chain reaction (PCR) molecular tests.^11^^–^^14^

On August 13, 2023, the Masaka Public Health Emergency Operations Center was notified by the District Surveillance Focal Person (Kyotera District) of six unexplained deaths that had occurred in three villages in Kabira subcounty. The strange deaths had occurred between August 6 and 10, 2023. Before death, the victims experienced various signs and symptoms, including fever, shortness of breath, abdominal pain, vomiting, loss of appetite, profuse sweating, swelling of the limbs, and body aches. The investigation initially considered alcohol poisoning as the cause of deaths; however, toxicology tests found normal methanol levels in adults who were tested. Postmortem examinations were inconclusive for those six deaths. As part of the Abbott Pandemic Defense Coalition^15^ mortuary surveillance study, blood and nasopharyngeal/oral/rectal swab specimens were collected from the deceased to determine the cause of unexplained deaths.

## MATERIALS AND METHODS

Venous blood was collected as well as nasopharyngeal/rectal swabs, cerebrospinal fluid, and tissue biopsies during the postmortem examinations. Autopsy specimens were sent to the Uganda Virus Research Institute (UVRI) to screen by PCR for Ebola, Marburg, Rift Valley Fever, and Crimean Congo Hemorrhagic Fever viruses.^16^ After the initial testing for hemorrhagic fevers, metagenomic next-generation sequencing (mNGS) testing of specimens was performed using the Illumina (San Diego, CA) DNA Prep (formerly Nextera XT) kit on the MiSeq NGS platform, and data were analyzed using an Abbott (Abbott Park, IL) bioinformatic research pipeline, DiVir.^17^

## RESULTS

**Figure 2.**
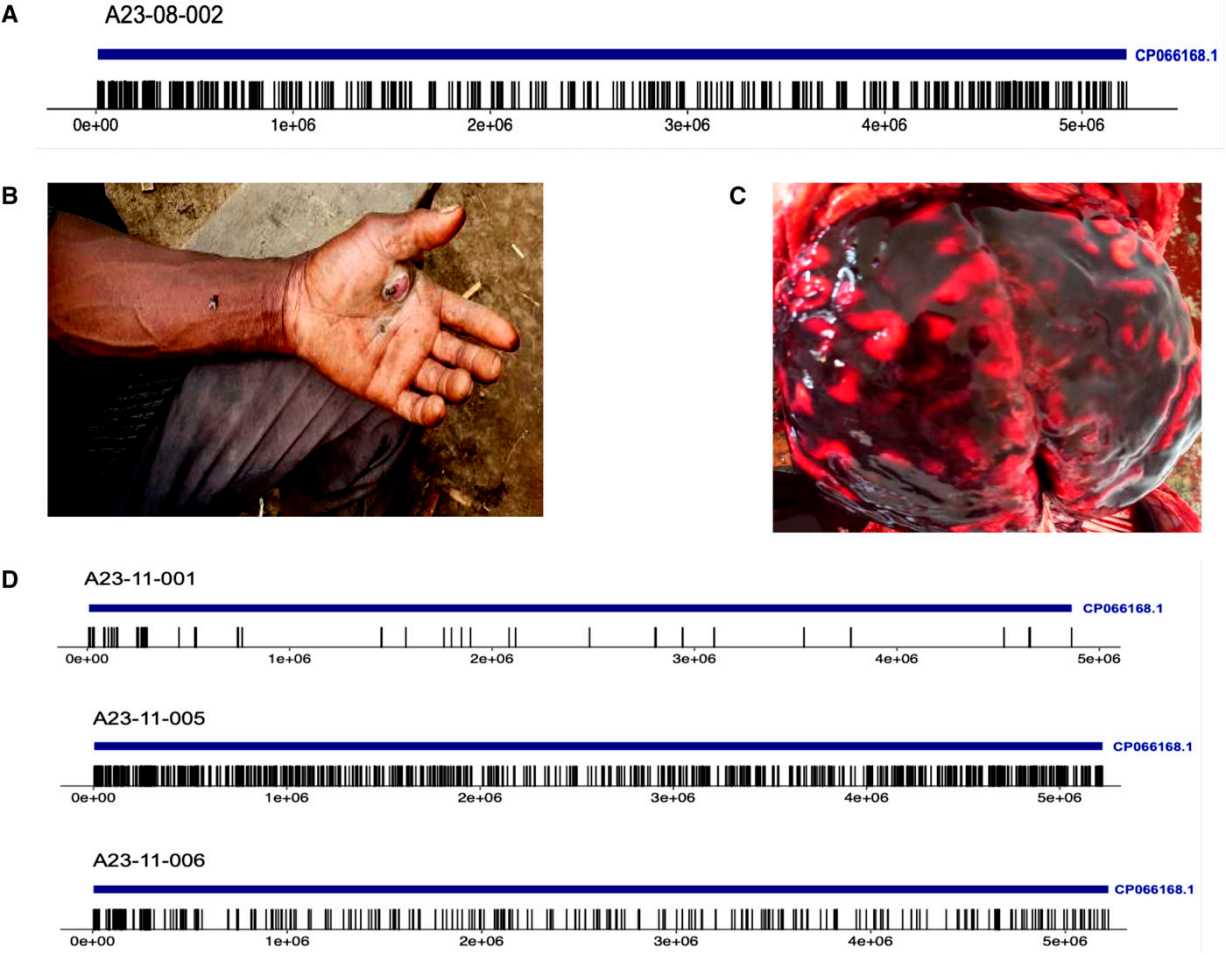
(**A**) Mapping and coverage plot for first sample deep sequenced in August 2023 and confirmed positive for *Bacillus anthracis*. The graph shows reads mapped to a *B. anthracis* reference genome (accession no. CP066168.1). (**B**) Skin ulcer or sore on the hand/arm with a black coloration or center was a common sign in most affected individuals before death. Other signs/symptoms included fever, vomiting, diarrhea, chest discomfort, difficulty in breathing, profuse sweating, and body aches. (**C**) Postmortem findings showed brain hemorrhage in some of the suspected cases that were later confirmed by metagenomic next-generation sequencing to have died of anthrax. Common to both cases was brain hemorrhage with increased intracranial pressure. (**D**) Mapping and coverage plots for additional samples deep sequenced in November 2023 and confirmed positive for *B. anthracis*. The graph shows reads mapped to a *B. anthracis* reference genome (accession no. CP066168.1) as in (**A**).

*Bacillus anthracis* reads were detected among the millions of mNGS sequences initially in only one patient (Figure 2A). This result was confirmed by an alternate target enrichment next-generation sequencing library preparation method, the Illumina Respiratory Pathogen ID/AMR Enrichment Panel, which contains specific probes for anthrax. Results were promptly communicated to the relevant authorities; however, with only one confirmed case, the cases were ruled unrelated, and different causes of death were given, including malaria, acute liver failure, and epileptic shock. Unexplained deaths continued to occur in Kyotera District during September and October 2023, with a total of 27 human deaths and 22 animal deaths. Before death, patients were reported to have varied manifestations, including arm swelling, blisters with exudate, skin lesions (Figure 2B), chest pain, difficulty in breathing, nonproductive cough, headache, and difficulty swallowing among other symptoms. It was reported that during a community dialogue meeting, family members recounted that some of the deceased had eaten beef from dead (diseased) cattle sold on the open market before their illness. Although it cannot be verified whether infection was acquired from eating beef or also through environmental exposure, symptoms were consistent with ingestion and inhalation anthrax. Between November 23 and 30, 2023, whole blood, nasopharyngeal/rectal swabs, cerebrospinal fluid, and other sample types were collected from six cadavers during postmortem examination of persons whose deaths were unexplained in Kyotera District. The first sample (Case 1) from a 48-year-old male farmer from Kyamakonkome village (Kabira County) was received at UVRI on November 23, 2023. The deceased had been taken to a traditional healer before his death.

Postmortem findings revealed yellowing (jaundice) on the dura mater and diffuse brain hemorrhage with increased intracranial pressure (narrowed sulci and flattened gyri) (Figure 2C). The lungs were enlarged with pulmonary edema and congestion. Similarly, for Case 2, the postmortem done on November 24, 2023 showed evidence of brain hemorrhage with features of increased intracranial pressure. Furthermore, the lungs were enlarged with pulmonary edema and congestion, but no emboli were observed.

The mNGS approach was used again to identify pathogens present in the samples (Figure 2D). The metagenomes generated for the six samples were analyzed using an in-house UVRI bioinformatics analysis pipeline, and *B. anthracis* was identified in all six specimens (Table 1). Results agreed with those obtained by Edge^18^ and DiVir^17^ bioinformatics pipelines. Sample aliquots were sent to the UVRI-Arua laboratories for anthrax-specific PCR testing,^13^^,^^19^ where three of six specimens tested positive for *B. anthracis* by an in-house PCR assay (Table 1).

**Table 1:** Summary of metagenomics sequencing and polymerase chain reaction results for seven postmortem samples from Kyotera

Case No.	Age (years)	Sex	District	Occupation	Signs and Symptoms before Death	Sample Collection Date	PCR UVRI-Arua	UVRI Metagenomics Pipeline	Edge Metagenomics Pipeline	DiVir Pipeline
1	57	Female	Kyotera	Farmer	Vomiting, nausea, diarrhea, loss of appetite, chest pain, muscle pain, joint pain, headache, and jaundice	August 14, 2023	N/A	–	–	*Bacillus anthracis*
2	48	Male	Kyotera	Farmer	Vomiting, nausea, diarrhea, intense fatigue, loss of appetite, chest pain, muscle pain, joint pain, headache, jaundice, and unconsciousness	November 23, 2023	Positive	*Bacillus anthracis*	*Bacillus anthracis*	*Bacillus anthracis*
3	63	Male	Kyotera	Farmer	Sudden death	November 24, 2023	Negative	*Bacillus anthracis*	*Bacillus anthracis*	*Bacillus anthracis*
4	65	Male	Kyotera	Farmer	Hemorrhagic clinical symptoms and difficulty breathing	November 25, 2023	Negative	*Bacillus anthracis*	*Bacillus anthracis*	*Bacillus anthracis*
5	37	Male	Kyotera	Farmer	Sudden death	November 25, 2023	Negative	*Bacillus anthracis*	*Bacillus anthracis*	*Bacillus anthracis*
6	35	Male	Kyotera	Farmer	Intense fatigue, muscle pain, and chest pain	November 29, 2023	Positive	*Bacillus anthracis*	*Bacillus anthracis*	*Bacillus anthracis*
7	34	Male	Kyotera	Farmer	Intense fatigue, joint pain, and body aches	November 30, 2023	Positive	*Bacillus anthracis*	*Bacillus anthracis*	*Bacillus anthracis*

N/A = not applicable; PCR = polymerase chain reaction; UVRI = Uganda Virus Research Institute.

## DISCUSSION

Twenty-seven deaths of unknown etiology were reported in Kyotera District (southern Uganda) between April and November 2023. Autopsy samples were collected during postmortems, and laboratory-based mNGS was performed to determine the cause of death. Deep sequencing identified the presence of *B. anthracis* reads in patient sequence libraries and confirmed an anthrax disease outbreak in Kyotera District. Initially, epidemiological data suggested that the deaths were unlikely to be related to each other. A review of subsequent cases, however, suggests that differential diagnosis by local clinicians was suboptimal and that identifying the outbreak cause earlier ought to have been possible without the need for mNGS.

Nevertheless, our findings demonstrate the potential benefits of unbiased metagenomic sequencing for identifying unknown causes of deaths. Postmortem samples were essential for confirming anthrax as the cause of death in Kyotera District. Laboratory test results also highlight the limitations of using PCR alone for confirming the etiology of disease outbreaks. False-negative PCR results could result from 1) low-level bacteremia below the detection limit of PCR, 2) sample quality-related issues that inhibit nucleic acid amplification, or 3) mutations present in the primer binding regions of the pathogen genome.

This is not the first time that similar deaths have occurred in Kyotera District. In December 2021, the Kyotera District Health Officer notified the Uganda Ministry of Health of 13 mysterious deaths in Kijonjo Parish, and the reasons for eight of those deaths were never determined.^20^ The eight cases had all sought care from traditional healers instead of going to health facilities. More recently, in November 2023 in Ibanda District of western Uganda, five people were hospitalized after eating meat suspected to be infected with *B. anthracis*. The district has since undertaken measures to control the spread of the disease.^21^

## CONCLUSION

There is an urgent need to gain a deeper understanding of the sociocultural factors associated with people not seeking care in local health facilities. Community sensitization campaigns are also critical in ensuring that people avoid eating meat from animals that have died of unknown causes or are diseased. Plans are underway to increase genomic surveillance in Kyotera District, to determine epidemiological linkages, and to scale up mortuary surveillance to other parts of the country. Furthermore, mNGS approaches should be incorporated as part of routine testing during suspected outbreaks of unknown etiology.

The sequence data presented in this study are available at https://github.com/UVRI-BCB/APDC/tree/main/Anthracis. All sequence data presented in this study can be obtained from the corresponding author upon reasonable request.
